# Formation of singlet oxygen in addition to hydroxyl radical via the Fenton reaction

**DOI:** 10.1016/j.redox.2025.103687

**Published:** 2025-05-17

**Authors:** Rino Shimizu, Haruki Watanabe, Sayaka Iida, Yorihiro Yamamoto, Akio Fujisawa

**Affiliations:** School of Bioscience and Biotechnology, Tokyo University of Technology, 1404-1 Katakura, Hachioji, Tokyo, 192-0982, Japan

**Keywords:** Fenton reaction, Singlet oxygen, Hydrogen peroxide

## Abstract

We established an LC-MS/MS method for detecting uric acid oxidation metabolites to evaluate reactive oxygen and nitrogen species, as uric acid gives specific products. Parabanic acid was identified during attempts to detect hydroxyl radical–specific products in the Fenton reaction. As parabanic acid is a singlet oxygen–specific product of uric acid, this indicates the Fenton system, which is known for the generation of hydroxyl radicals, also forms singlet oxygen products. This notion was confirmed by replacing uric acid with tryptophan, which resulted in the formation of singlet oxygen–specific oxidation products (*cis*- and *trans*-WOOH) and their reductants, *cis*- and *trans*-WOH. Product amounts were reduced in a dose-dependent manner by the addition of the singlet oxygen quenchers sodium azide or 1,4-diazabicyclo[2.2.2]octane. Surprisingly, the estimated amount of singlet oxygen produced was 50- to 70-fold greater than that of hydroxyl radical, considering the quantum yield of the reaction between uric acid and singlet oxygen. The formation of singlet oxygen under anaerobic conditions suggested it was derived from hydrogen peroxide. The production of non-labeled parabanic acid, even in an ^18^O_2_ atmosphere or the presence of H_2_^18^O, supported this hypothesis. These results confirmed that singlet oxygen was derived from hydrogen peroxide. The proposed mechanism of singlet oxygen formation is as follows. Two hydrogen peroxyl radicals formed by the reaction of hydrogen peroxide and ferric ion or hydroxyl radical are coupled to form a hydrogen tetraoxide, which subsequently decomposes to form singlet oxygen and hydrogen peroxide via a Russell-like mechanism. Finally, it was observed that significantly more singlet oxygen was generated in whole human blood compared with red blood cell–depleted blood during pseudo-inflammation initiated by lipopolysaccharide addition, suggesting that singlet oxygen formation was due to the Fenton reaction. Thus, the Fenton reaction may be a novel pathway for singlet oxygen production.

## Highlights

• The Fenton reaction generates a dominant amount of singlet oxygen.
• That singlet oxygen is derived from hydrogen peroxide, not oxygen molecules.
• That singlet oxygen was produced from the decomposition of hydrogen tetraoxide.
• That singlet oxygen can be generated in inflammatory conditions.
• The Fenton reaction should be reconsidered as a singlet oxygen-producing system.

## Graphical abstract

**Figure:**
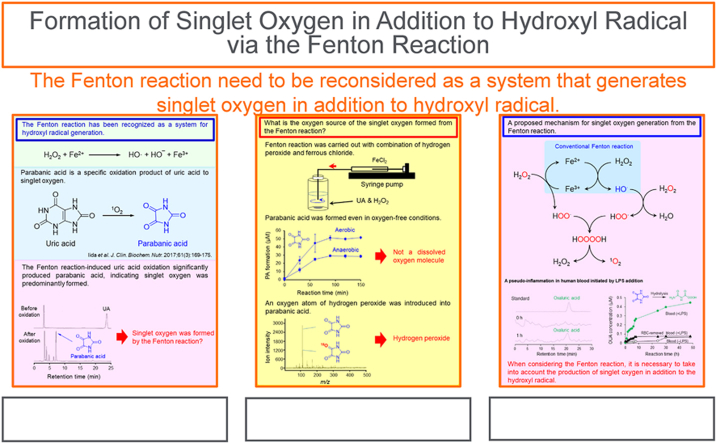

## Abbreviations

DABCO: 1,4-diazabicyclo[2,2,2]octane
DIAA: (2,5-dioxoimidazolidin-4-ylidene)aminocarbonylcarbamic acid
LPS: lipopolysaccharide
MS/MS: tandem mass spectrometry
TOFMS: time-of-flight mass spectrometry
PA: parabanic acid
UA: uric acid

## 1 Introduction

Reactive oxygen species (ROS) and reactive nitrogen species (RNS) play important roles in immune defense [[1], [2], [3]] and redox signaling [[4], [5], [6]] *in vivo*. However, excessive ROS and RNS production leads to oxidative stress, which is associated with a variety of diseases involving oxidative damage of cells, tissues, and organs [[7], [8], [9], [10]]. Hydroxyl radical (HO•) is recognized as the most reactive ROS. Because of its high reactivity, hydroxyl radical is thought to cause significant oxidative damage to biological molecules such as DNA [[11], [12], [13]], lipids [[14], [15], [16], [17]], and proteins [[18], [19], [20], [21]]. The Fenton reaction is the most significant system that should be considered as a generator of hydroxyl radicals *in vivo*.

The Fenton reaction, which involves the reduction of hydrogen peroxide (H_2_O_2_) by a transition metal ion, is a well–characterized pathway for hydroxyl radical formation. It is believed that the Fenton reaction initiates oxidative stress *in vivo*. For example, ferrous ion (Fe^2+^) reduces H_2_O_2_ to form a hydroxyl radical and HO^−^ via the Fenton reaction, in the process converting Fe^2+^ into ferric ion (Fe^3+^). The Fenton reaction has been extensively studied for its roles in mediating oxidative stress and cellular damage. The reaction can be summarized by the following chemical equation:

(Eq.1) H_2_O_2_+Fe^2+^→HO• + HO^−^ + Fe^3+^

Oxidative damage caused by the Fenton reaction is a major contributor to several pathological conditions, including neurodegenerative diseases [[22], [23], [24], [25]], cancer [[26], [27], [28], [29]], and cardiovascular disorders [[30], [31], [32], [33]].

The Fenton reaction is thought to play an extremely important role in ferroptosis. Ferroptosis is a recently identified form of regulated cell death fundamentally linked to iron and characterized by lipid peroxidation [[34], [35], [36]]. Unlike apoptosis or necrosis, ferroptosis is dependent upon on the iron–catalyzed formation of lipid hydroperoxides, which cause damage to the membrane that ultimately leads to cell death [[37], [38], [39]]. This process is exacerbated by the inhibition of glutathione peroxidase 4 (GPX4), an enzyme responsible for reducing lipid peroxides [[40], [41], [42]].

Intracellular iron pools, particularly redox–active ferrous iron, amplify lipid peroxidation via the Fenton reaction. Hydroxyl radicals produced by this reaction oxidize polyunsaturated fatty acids in the cell membrane, which triggers ferroptotic pathways [[43], [44], [45], [46]]. Thus, the Fenton reaction serves as a critical mediator of ferroptosis, linking iron metabolism to oxidative stress–induced cell death.

The interplay between the Fenton reaction and ferroptosis has profound implications for human health. Iron accumulation and oxidative stress are hallmark features of neurodegenerative diseases such as Parkinson's and Alzheimer's, contributing to neuronal loss via ferroptosis–like mechanisms [[47], [48], [49], [50], [51]]. Similarly, in cancer, ferroptosis represents both a vulnerability and a defense mechanism. Although tumor cells often exploit iron to facilitate rapid proliferation, they are also susceptible to ferroptotic death, providing a potential therapeutic target [[52], [53], [54], [55]]. Emerging evidence also implicates the Fenton reaction and ferroptosis in conditions such as ischemia–reperfusion injury [[56], [57], [58]], in which oxidative stress plays a central role.

As discussed above, the Fenton reaction is recognized as a conventional hydroxyl radical–producing system. However, considerable attention is now focusing on elucidating the generation of singlet oxygen *in vivo*. Umeno et al. reported that singlet oxygen–induced linoleic acid oxidation products are useful markers for the early detection of type 2 diabetes, as singlet oxygen may be involved in the onset of the disease [59,60]. They also reported that singlet oxygen plays an important role in the exacerbation of bronchial asthma via nerve growth factor production during inflammation [61]. Stanley et al. reported that indoleamine 2,3-dioxygenase oxidizes tryptophan (Trp) into (2*S*,3a*R*,8a*R*)-3a-hydroperoxy-1,2,3,3a,8,8a-hexahydropyrrolo[2,3-*b*]indole-2-carboxylic acid (*cis*-WOOH) using singlet oxygen in the presence of hydrogen peroxide. In turn, *cis*-WOOH shows strong artery–relaxing effects [62].

Singlet oxygen also causes lipid peroxidation. Furthermore, although radical species only induce oxidation reactions in highly unsaturated fatty acids, singlet oxygen also oxidizes monoenoic acids via the ene–reaction. The rate constant of the reaction between singlet oxygen and an alkene is estimated at approximately 10^5^–10^6^ M^−1^s^−1^ [63], greater than that of peroxyl radical and alkadiene (∼10^2^ M^−1^s^−1^) [64]. Thus, it is believed that singlet oxygen, if generated *in vivo*, is sufficient to cause lipid peroxidation.

In this study, we examined the oxidation of uric acid (UA) initiated by hydroxyl radical. In humans, UA is a terminal metabolite of purines and an essential water-soluble antioxidant. UA reacts with ROS to yield various ROS–specific oxidation products, and free radical–, singlet oxygen–, hypochlorous ion–, and peroxynitrite–induced oxidation leads to the formation of allantoin [65], parabanic acid (PA) and its hydrolysate oxaluric acid (OUA) [66,67], 5-*N*-carboxyimino-6-*N*-chloroaminopyrimidine-2,4(3*H*)-dione [68], and triuret [69], respectively. We recently established a method for analyzing these UA oxidation products using LC/MS/MS for identification of ROS and RNS generated *in vivo* [70]. In order to elucidate the mechanism of allantoin production via free radical–initiated UA oxidation, UA was oxidized with hydroxyl radicals generated through the Fenton reaction of hydrogen peroxide and ferrous ion. In this reaction process, the formation of PA was observed, suggesting that singlet oxygen was produced via the Fenton system. The formation of singlet oxygen was confirmed by replacement of UA with Trp, which resulted in the formation of another singlet oxygen–specific oxidation product, *cis*-WOOH and its 3a,8a-epimer *trans*-WOOH [62], as well as their reductants, *cis*- and *trans*-WOH. Considering that most of the singlet oxygen would be physically quenched and that the ratio of singlet oxygen to UA reacted would be < 0.01, [66] the amount of singlet oxygen produced was estimated at >70 % of the eliminated hydrogen peroxide, indicating that singlet oxygen was the dominant species generated by the Fenton reaction. These processes will be discussed in greater detail below, but we believe the mechanism of singlet oxygen formation in the Fenton reaction involves hydrogen tetraoxide (HOOOOH), with coupling of the products of two hydrogen peroxyl radicals (HOO•) formed during the Fenton reaction.

Based on our present results, we thus believe the results of previous studies related to the Fenton reaction should be seriously reconsidered.

## 2 Materials and methods

### 2.1 Chemicals

PA, UA, Trp, tyrosine (Tyr), 3,4-dihydroxyphenylalanine (HO-tyr), H_2_O_2_, FeCl_2_, NaN_3_, 1,4-diazabicyclo[2.2.2]octane (DABCO), and other chemicals were purchased from Fujifilm Wako Pure Chemical Co. (Osaka, Japan) or Tokyo Chemical Industry Co., Ltd. (Tokyo, Japan), and used as received. H_2_O_2_ labeled with ^18^O_2_ was purchased from ICON isotopes (Summit, NJ, USA). OUA, a hydrolysate of PA, was prepared by hydrolysis of PA using aqueous ammonia. Both *trans*- and *cis*-WOH were kindly provided by Professor Roland Stocker.

### 2.2 UA, Trp, and Tyr oxidation via the Fenton reaction

Oxidation of UA and Trp by ROS generated via the Fenton reaction was analyzed. The Fenton reaction was initiated by addition of FeCl_2_ to H_2_O_2_. H_2_O_2_ (final concentration 10 mM) and UA (200 μM) or Trp (1.0 mM) were dissolved in phosphate buffer (40 mM, pH 7.4) with/without the ^1^O_2_ quencher NaN_3_ or DABCO. FeCl_2_ aqueous solution was gradually added to the UA solution at a constant rate of 1.7 μM/min for 150 min. During analysis of UA oxidation under anaerobic conditions, the solution was bubbled with N_2_ gas for 1 h before the reaction, as described previously [67]. Changes in the concentrations of UA and its oxidation products were determined using high-performance liquid chromatography (HPLC), as described below.

Dissolved oxygen was removed by bubbling samples with N_2_ gas. The aqueous reaction mixture was poured into an airtight vial connected to a N_2_ gas cylinder and vacuum pump. N_2_ gas was then delivered into the well-stirred solution with vigorous bubbling for 3 h. For analysis of oxidation in the presence of ^18^O_2_, ^18^O_2_ gas was introduced into the vial using a vacuum pump. Removal of dissolved oxygen using this method was confirmed previously [67].

For analysis of Tyr oxidation, Tyr (1.0 mM) was dissolved in 1.0 mL of H_2_O or H_2_^18^O, and 5 μL of FeCl_2_ (20 mM) aqueous solution was added every 20 min for 120 min.

### 2.3 Pseudo-inflammation in human blood initiated by lipopolysaccharide (LPS) addition

To investigate the formation of singlet oxygen *in vivo*, pseudo–inflammation was induced. Blood was taken from a healthy volunteer using heparin as an anticoagulant. Red blood cell (RBC)–depleted blood was then prepared as follows. Collected blood was centrifuged (26,200×*g*, 5 min) to separate plasma, white blood cells, and RBCs, and the RBCs were removed using an aspirator. Lipopolysaccharide (LPS) (2.5 μg/mL) was added to the intact and RBC–depleted blood samples, which were then incubated at 37 °C for 48 h. During incubation, samples were collected hourly from 0 to 8 h and again at 24, 30, and 48 h after LPS addition. These samples were centrifuged (26,200×*g*, 5 min) to separate plasma as the supernatant. The plasma was collected and added to a double volume of water and subsequently a double volume of methanol. The plasma was shaken vigorously and centrifuged (26,200×*g*, 10 min) to separate insoluble precipitates. Next, 5 μL of the supernatant was analyzed using HPLC and tandem mass spectrometry (LC-MS/MS) using a method optimized for the analysis of OUA, as described below.

### 2.4 HPLC-UV analysis

Changes in the concentrations of UA, its oxidation products, and H_2_O_2_ were analyzed using reverse-phase HPLC with ultraviolet (UV) detection (HPLC-UV). Aqueous formic acid (adjusted to pH 3.0) was delivered at 1.0 mL/min as a mobile phase, and a Develosil C30-UG column (5 μm, 4.6 mm × 250 mm, Nomura Chemical Co. Ltd., Tokyo, Japan) was used for separation. Products were detected by monitoring the absorption of the eluate at 210 nm. Changes in the concentrations of Tyr and its oxidation products were also analyzed using HPLC-UV.

### 2.5 LC-TOFMS analysis

Accurate mass-to-charge ratio (*m/z*) values for PA and (2,5-dioxoimidazolidin-4-ylidene)aminocarbonylcarbamic acid (DIAA) were obtained using an optimized LC–time-of-flight mass spectrometry (TOFMS) system. All acquired *m/z* values were compensated with trifluoroacetic acid as an internal standard**.** The mobile phase was aqueous formic acid (pH 3.0) delivered at 1.0 mL/min, and a Develosil C30-UG (5 μm, 4.6 mm × 250 mm, Nomura Chemical Co., Ltd.) column was used for separation. One-fourth of the mobile phase flow was introduced into the ionizing chamber of the TOFMS, and positive- and negative-mode electrospray ionization (ESI) was carried out at ionization potentials of +2500 V and −2000 V, respectively. Applied voltages to the ring lens, outer orifice, inner orifice, and ion guide were set to +5 V, +30 V, +5 V, and +500 V in positive mode and −5 V, −20 V, −5 V, and −500 V in negative mode, respectively.

### 2.6 LC-MS/MS analysis

LC-MS/MS was used to measure the amount of OUA formed. Aqueous formic acid (adjusted to pH 3.0) was used as a mobile phase delivered at 0.2 mL/min. A Develosil C30-UG column (5 μm, 2.0 mm × 250 mm, Nomura Chemical Co. Ltd.) was used for separation at room temperature. Negative ionization was carried out at an ionization potential of −3.2 kV using an electrospray probe. Multiple reaction monitoring was used for identification and quantification. The optimized combination of product and precursor ions for OUA was determined as −59/–131.

## 3 Results

### 3.1 PA formation during UA oxidation induced by H_2_O_2_ and FeCl_2_ addition under aerobic and anaerobic conditions

**Fig. 1:**
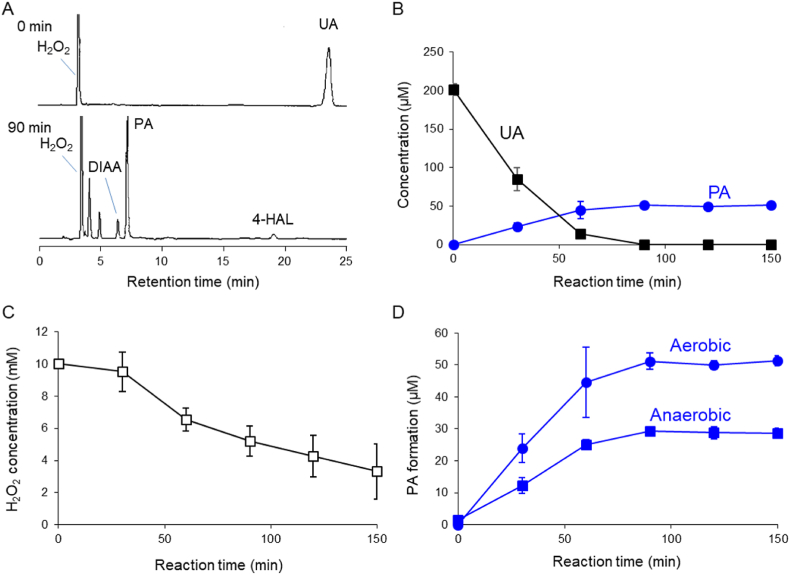
Oxidation of 200 μM UA in the presence of 10 mM H_2_O_2_ induced by the addition of FeCl_2_ (1.7 μM/min) for 150 min. (A) HPLC chromatograms of reaction mixtures at 0 min (upper) and 90 min (lower) after introduction of FeCl_2_ solution. (B) Time course of changes in concentrations of UA (■) and PA (●) during Fenton reaction–induced UA oxidation. (C) H_2_O_2_ degradation during Fenton reaction–induced UA oxidation. (D) Time course of PA formation under aerobic (●) and anaerobic (■) conditions. All data are expressed as the mean ± SD (*n* = 3).

We analyzed the oxidation of UA by ROS generated via the Fenton reaction of FeCl_2_ and H_2_O_2_. FeCl_2_ was added to phosphate buffer solution (40 mM, pH 7.4) containing UA (200 μM) and H_2_O_2_ (10 mM) at a constant rate (1.7 μM/min) for 150 min. The production of PA and its precursors 4-hydroxyallantoin (4-HAL) and DIAA was analyzed by HPLC 90 min after FeCl_2_ addition (Fig. 1A). PA formation coincided with UA degradation, and the yield of PA reached 25.5 % (Fig. 1B). H_2_O_2_ was also detected (Fig. 1A), and the concentration decreased as FeCl_2_ was added, with a decrement after 150 min of approximately 6.7 mM (Fig. 1C). Formation of PA was observed under both aerobic and anaerobic conditions (Fig. 1D).

### 3.2 Inhibition of PA formation by NaN_3_ and DABCO

**Fig. 2:**
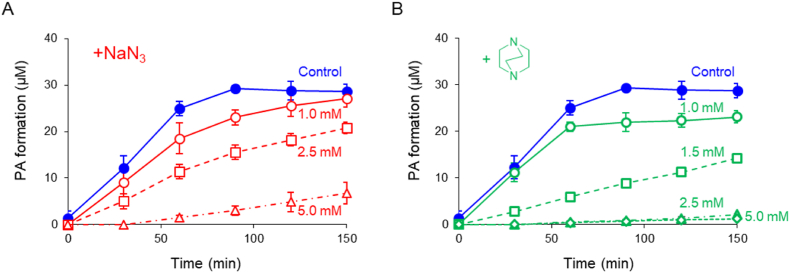
Time courses of PA formation via Fenton reaction–induced UA oxidation and its inhibition using authentic ^1^O_2_ quenchers. To a reaction mixture containing UA (200 μM) and H_2_O_2_ (1.0 mM), FeCl_2_ was introduced at a constant rate (1.7 μM/min). Inhibition by addition of 1.0 mM (○), 2.5 mM (☐), and 5.0 mM (△) NaN_3_ (A) and 1.0 mM (○), 1.5 mM (☐), 2.5 mM (△), and 5.0 mM (◇) DABCO (B). All data are expressed as the mean ± SD (*n* = 3).

The conventional singlet oxygen quenchers NaN_3_ and DABCO were added to separate reaction solutions to determine whether PA production was associated with singlet oxygen. Production of PA was significantly inhibited in a dose-dependent manner by the addition of NaN_3_ (Fig. 2A) or DABCO (Fig. 2B).

Formation of *cis*- and *trans*-WOH during oxidation of Trp in the Fenton reaction and inhibition of *cis*- and *trans*-WOH formation by NaN_3_ and DABCO.

**Fig. 3:**
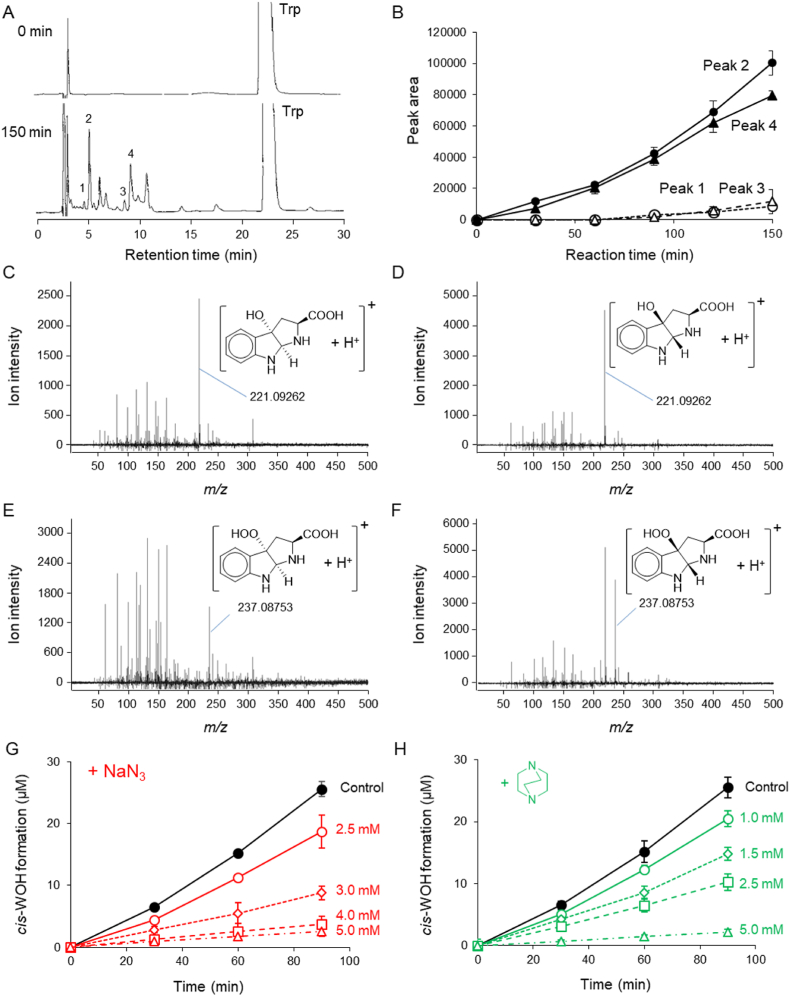
Production of WOHs and WOOHs via Fenton reaction–induced Trp oxidation. To a reaction mixture containing Trp (1.0 mM) and H_2_O_2_ (10 mM), FeCl_2_ was introduced at a constant rate (1.7 μM/min). (A) HPLC chromatograms of reaction mixtures at 0 min (upper) and 30 min (lower). (B) Time course of changes in areas of peak 1 (○), peak 2 (●), peak 3 (△), and peak 4 (▲). Data are expressed as the mean ± SD (*n* = 3). MS spectra of peak 1 (C), peak 2 (D), peak 3 (E), and peak 4 (F) acquired using LC-TOFMS in positive ESI mode. Inhibition of *cis*-WOH formation by the addition of 2.5 (○), 3.0 (◇), 4.0 (□), and 5.0 mM (△) NaN_3_ (G) or 1.0 (○), 1.5 (◇), 2.5 (□), and 5.0 mM (△) DABCO (H).

Trp oxidized via the Fenton reaction was analyzed using HPLC-UV. The chromatogram of a 30-min reaction revealed four peaks (nos. 1–4) (Fig. 3A), the intensity of which increased with increasing reaction time (Fig. 3B). The retention times of peaks 1 and 2 were identical to those of authentic *trans*- and *cis*-WOH, respectively. The products contained in the eluates of these peaks were analyzed using LC-TOFMS with positive ESI. The MS spectra of peaks 1 and 2 (Fig. 3C and D, respectively) revealed dominant ions with accurate *m/z* values of +221.09382 and + 221.09411, respectively. The accurate *m/z* values of the dominant ions in MS spectra of peaks 3 and 4 (Fig. 3E and F, respectively) were +237.08813 and + 237.08825, respectively, indicating that these products were *trans*- and *cis*-WOOH. The increments of these peak areas were also significantly reduced by the addition of NaN_3_ or DABCO. The concentration of *cis*-WOH (peak 2) was significantly reduced in a dose-dependent manner by the addition of NaN_3_ (Fig. 3G) or DABCO (Fig. 3H).

Formation of PA under ^18^O_2_ atmospheric conditions or in H_2_^18^O

**Fig. 4:**
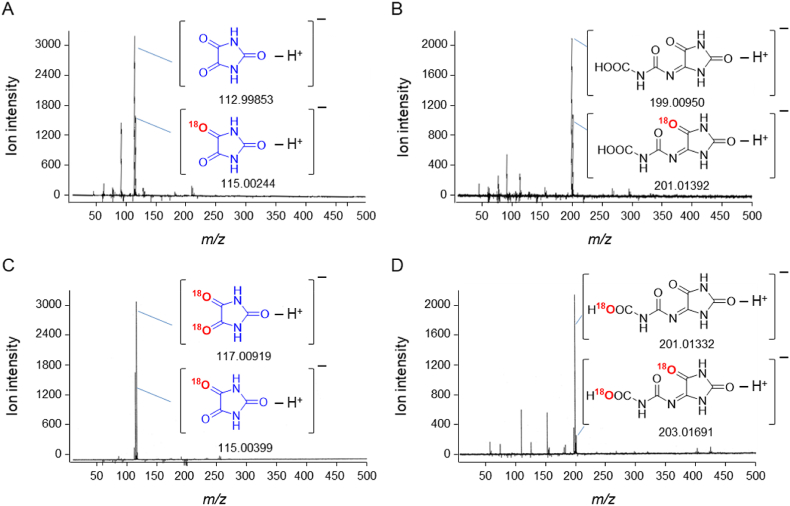
PA and DIAA formation via Fenton reaction–initiated UA oxidation in an ^18^O_2_ atmosphere and in H_2_^18^O. MS spectra of PA (A) and its precursor DIAA (B) formed in an ^18^O_2_ atmosphere. MS spectra of PA (C) and DIAA (D) produced in H_2_^18^O. All MS spectra were acquired using LC-TOFMS in negative ESI mode.

PA was formed even under anaerobic conditions (Fig. 1D). To confirm that the singlet oxygen is not derived from dissolved molecular oxygen, UA was oxidized in an ^18^O_2_ atmosphere. Oxidation was initiated via the Fenton reaction, and the formation of PA was analyzed using negative-mode LC-TOFMS. MS spectra of PA (Fig. 4A) and its direct precursor, DIAA (Fig. 4B), were acquired. Non-labeled (accurate *m/z* −112.99853, theoretical −112.99872) and mono–^18^O-labeled PA (accurate *m/z* −115.00244, theoretical −115.00296) and the corresponding non-labeled (accurate *m/z* −199.00950, theoretical −199.01034) and mono–^18^O-labeled DIAA (accurate *m/z* −201.01392, theoretical −201.01459) ions were detected.

UA was then oxidized in H_2_^18^O. Mono–^18^O-labeled PA (accurate *m/z* −115.00399) and di–^18^O-labeled PA (accurate *m/z* −117.00919, theoretical −117.00721) were formed (Fig. 4C). Similarly, mono–^18^O-labeled DIAA (accurate *m/z* −201.01332, theoretical *m/z* −201.01514) and di–^18^O-labeled DIAA (accurate *m/z* −203.01691, theoretical *m/z* −203.01884) were produced (Fig. 4D).

Oxidation of UA initiated with H_2_^18^O_2_ and Fe^2+^

**Fig. 5:**
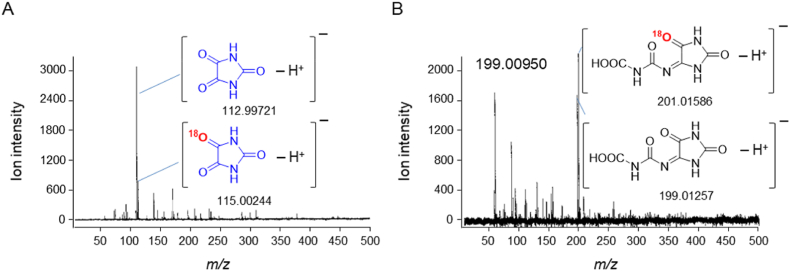
PA and DIAA formation via Fenton reaction–initiated UA oxidation using H_2_^18^O_2_ in non-labeled water and air. MS spectra of PA (A) and DIAA (B) acquired using LC-TOFMS in negative ESI mode.

To identify the source of singlet oxygen generated in the Fenton reaction, we examined the oxidation of UA initiated with H_2_^18^O_2_ and FeCl_2_ in non-labeled water under normal atmospheric conditions. MS spectra of PA (Fig. 5A) and DIAA (Fig. 5B) were acquired by LC-TOFMS. In addition to non-labeled PA (accurate *m/z* −112.99721) and DIAA (accurate *m/z* −199.01257), mono–^18^O-labeled PA (accurate *m/z* −115.00244) and mono–^18^O-labeled DIAA (accurate *m/z* −201.01586) were detected.

### 3.3 Reaction of hydroxyl radical and water

**Fig. 6:**
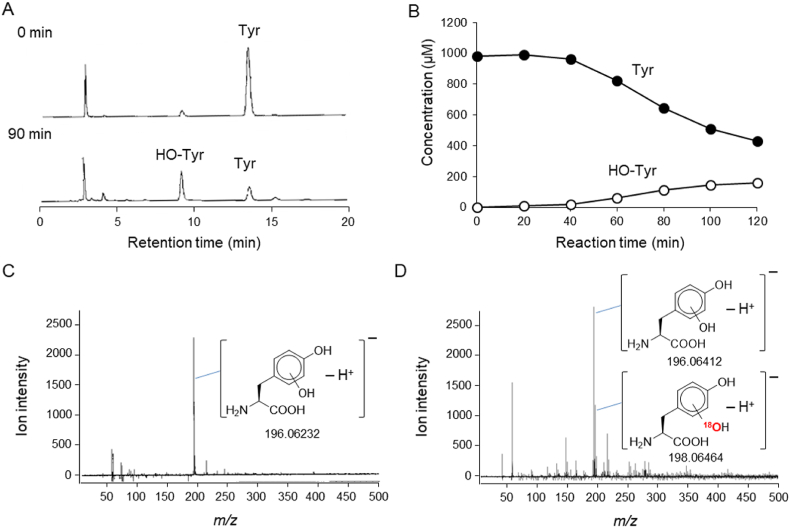
Tyr hydroxylation by HO· generated via the Fenton reaction. To a reaction mixture containing Tyr (1.0 mM) and H_2_O_2_ (10 mM), FeCl_2_ was introduced at a constant rate (1.7 μM/min). (A) HPLC chromatograms of reaction mixtures at 0 min (upper) and 90 min (lower). (B) Time course of changes in the concentrations of HO-Tyr (○) and Tyr (●) during Fenton reaction–induced oxidation. (C) MS spectrum of HO-Tyr formed in non-labeled H_2_O. (D) MS spectrum of HO-Tyr formed in H_2_^18^O.

To determine whether hydroxyl radical reacts with H_2_O to form another hydroxyl radical, we oxidized Tyr via the Fenton reaction in H_2_O or H_2_^18^O. A peak thought to be hydroxy-tyrosine (HO-Tyr) was detected on the HPLC chromatogram (Fig. 6A), and the intensity of this peak increased with Tyr degradation (Fig. 6B). The MS spectrum was confirmed using LC-TOFMS in negative ESI mode (Fig. 6C). A typical ion showing an accurate *m/z* value of −196.06232 was observed. This ion was thought to be a deprotonated ion of HO-Tyr (theoretical *m/z* −196.06098). Tyr was then oxidized in H_2_^18^O, and the formation of HO-Tyr was analyzed using MS (Fig. 6D). In addition to non-labeled HO-Tyr (accurate *m/z* −196.06412), mono–^18^O-labeled HO-Tyr deprotonated ion (accurate *m/z* −198.06464, theoretical *m/z* −198.06523) was observed.

### 3.4 Singlet oxygen formation during LPS-induced pseudo-inflammation in human blood

**Fig. 7:**
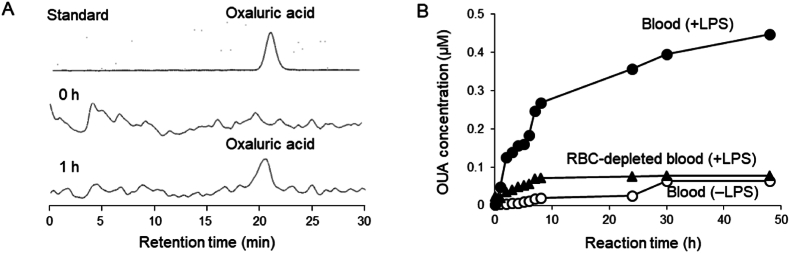
Detection of OUA and change in OUA plasma concentration during LPS-induced pseudo-inflammation in human blood. (A) Chromatograms of LC/MS/MS analyses of OUA standard (upper), blood at 0 h after LPS addition (middle), and 1 h after LPS addition (lower). (B) Time course of the change in OUA concentration in blood with LPS addition (●), control blood (○), and RBC-depleted blood with LPS addition (▲).

To examine singlet oxygen formation under *in vivo*–mimicking conditions, LPS-induced pseudo-inflammation in human blood was analyzed. LPS (2.5 μg/mL) was added to whole human blood and RBC-depleted blood and incubated at 37 °C for 48 h, and the change in the concentration of OUA, a hydrolysate of PA, was measured using the optimized LC-MS/MS method. OUA was detected in LPS-containing human blood 1 h after addition (Fig. 7A), and the OUA concentration increased significantly during incubation. By contrast, no significant change in OUA concentration was observed in the absence of LPS or in RBC-depleted blood (Fig. 7B).

## 4 Discussion

In the current study, we demonstrated that singlet oxygen was predominantly produced via the Fenton reaction, which is considered a conventional hydroxyl radical–generating system. We found that PA and its precursor, DIAA, were formed during UA oxidation induced by the Fenton reaction of H_2_O_2_ and Fe^2+^ (Fig. 1A). As both compounds were identified to be singlet oxygen–specific oxidation products [66,67], it was suggested that singlet oxygen was generated through the Fenton reaction. The concentration of PA reached approximately 50 μM when 200 μM UA was oxidized (Fig. 1B). As the molecular yield (defined as the ratio of the amounts of reacted– to formed–singlet oxygen) in the reaction with UA was thought to be <1.0 % [66], the concentration of singlet oxygen produced was estimated to be > 5.0 mM. Consumption of H_2_O_2_ reached approximately 6.7 mM (Fig. 1C), suggesting that most of the consumed H_2_O_2_ was converted into singlet oxygen. Inhibition of PA formation using conventional singlet oxygen quenchers was also examined. Addition of NaN_3_ or DABCO significantly reduced the production of PA in a dose-dependent manner (Fig. 2A and B). These results indicate that the formation of PA via Fenton reaction–induced UA oxidation was due to singlet oxygen.

We also oxidized Trp via the Fenton reaction. Stanley et al. reported that singlet oxygen–induced Trp oxidation produces *trans*- and *cis*-WOOH [62], which are thought to be converted into corresponding *trans*- and *cis*-WOH via Fe^2+^–induced decomposition of hydroperoxide. Indeed, the area of HPLC peaks 1–4 increased as Trp decreased during Fenton reaction–induced Trp oxidation (Fig. 3A and B). To determine whether these peaks were singlet oxygen–specific Trp oxidation products, MS spectra were acquired in positive ESI mode (Fig. 3C–F). Fig. 3C and D shows MS spectra of peaks 1 and 2, respectively. Accurate *m/z* values of the dominant ions (peak 1 + 221.09382, peak 2 + 221.09411) suggested that they have a similar chemical formula, C_11_H_13_N_2_O_3_ (theoretical *m/z* +221.09262), identical to that of *trans*- and *cis*-WOH protonated ions. As the MS spectra and chromatographic retention times of *trans*- and *cis*-WOH authentic standards were also identical to those of peaks 1 and 2 (data not shown), peaks 1 and 2 were identified as *trans*- and *cis*-WOH, respectively. MS spectra of peaks 3 and 4 were also acquired. Similar dominant ions with an *m/z* value of +237 were detected in both MS spectra (Fig. 3E and F), and the corresponding accurate *m/z* values were +237.08813 and + 237.08825, respectively. Based on these *m/z* values, peaks 3 and 4 were postulated to be *trans*- and *cis*-WOOH (theoretical *m/z* +237.08753), respectively. The formation of these singlet oxygen–specific Trp oxidation products was also suppressed by the addition of ^1^O_2_ quenchers, similar to PA formation. The time courses of typical production of *cis*-WOH and its inhibition by the addition of NaN_3_ (Fig. 3G) or DABCO (Fig. 3H) were also analyzed. Both quenchers significantly inhibited the formation of *cis*-WOH in a dose-dependent manner. Taken together, these results strongly suggest that singlet oxygen was formed via the Fenton reaction of H_2_O_2_ and Fe^2+^.

To identify the source of the oxygen atom of singlet oxygen formed in the Fenton reaction, we conducted experiments under oxygen–free and ^18^O_2_ atmosphere conditions, in H_2_^18^O solution, and using H_2_^18^O_2_ instead of H_2_O_2_. Oxygen–free analyses were conducted by bubbling samples with N_2_ gas for 3 h before oxidation of UA. Interestingly, PA formed even under anaerobic conditions (Fig. 1D), indicating that the singlet oxygen produced was not derived from dissolved oxygen. The formation of non–^18^O-labeled PA and the corresponding formation of non–^18^O-labeled DIAA, a precursor in PA production, in an ^18^O_2_ atmosphere supported this conclusion (Fig. 4A and B).

**Scheme 1:**
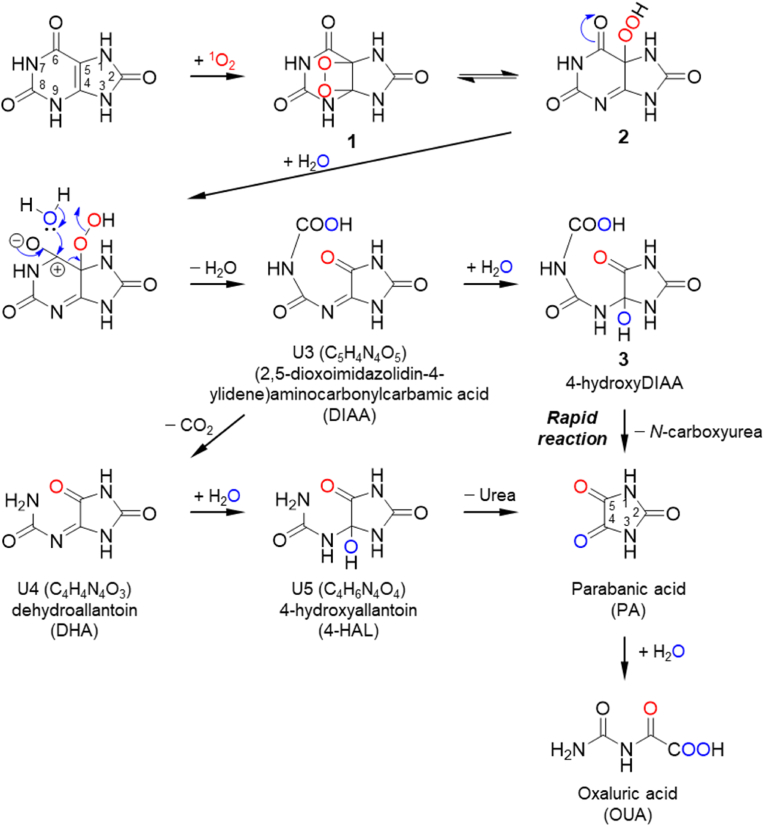
Mechanism for reaction of UA and singlet oxygen.

We previously demonstrated that one of the two extra oxygen atoms introduced into PA is derived from singlet oxygen and the other from water (Scheme 1) [67]. This result indicated that non-labeled singlet oxygen is formed during the Fenton reaction. Therefore, the singlet oxygen formed in the Fenton reaction in this study was derived from H_2_O_2_ or water rather than dissolved oxygen. However, small amounts of mono–^18^O-labeled PA and DIAA were formed. It is believed that energy exchange between singlet-state oxygen (^1^O_2_) and triplet-state oxygen (^3^O_2_) occurs to form another singlet oxygen molecule. If this energy exchange reaction occurs, the observed life-span of singlet oxygen could be prolonged in the presence of dissolved molecular oxygen. Indeed, the amount of PA formed under aerobic conditions was greater than the amount formed under anaerobic conditions (Fig. 1D). Taken together, these data indicate that energy transfer from singlet-state ^16^O_2_ ([16][^1^O_2_]) to triplet-state ^18^O_2_ ([18][^3^O_2_]) produces singlet-state ^18^O_2_ ([18][^1^O_2_]) in an ^18^O_2_ atmosphere (Eq. (2)):

(Eq. 2) ^16^[^1^O_2_] + ^18^[^3^O_2_]→^16^[^3^O_2_] + ^18^[^1^O_2_]

Therefore, the formation of mono–^18^O-labeled PA in an ^18^O_2_ atmosphere can be attributed to the formation of ^18^[^1^O_2_].

We also examined the oxidation of UA in H_2_^18^O. This reaction led to the formation of mono–^18^O-labeled PA and DIAA (Fig. 4C and D) and indicated that non-labeled singlet oxygen forms because a single oxygen atom derived from H_2_O is introduced into PA or DIAA**.** Hence, these results strongly suggest that the singlet oxygen observed in this study was produced from H_2_O_2_. To confirm that the singlet oxygen formed was derived from H_2_O_2_, we oxidized UA using H_2_^18^O_2_ instead of H_2_O_2_ in normal H_2_O under ordinary atmospheric conditions. Production of both mono–^18^O-labeled PA (Fig. 5A) and DIAA (Fig. 5B) was observed, confirming the generation of ^18^O-labeled singlet oxygen.

The following mechanism is proposed to explain the predominant generation of singlet oxygen in the Fenton reaction between H_2_O_2_ and Fe^2+^. H_2_O_2_ reacts with Fe^2+^ to form hydroxyl radical, HO^−^, and Fe^3+^ (Eq. (1)). The subsequent reaction between H_2_O_2_ and Fe^3+^ leads to the production of hydrogen peroxyl radical (HOO•) and Fe^2+^ (Eq. (3)). Moreover, the hydroxyl radical formed absorbs a hydrogen atom from H_2_O_2_ to also produce hydrogen peroxyl radical (Eq. (4)). It has been reported that in the presence of UA, Fe^3+^ forms a loose complex with UA, which lowers the reduction potential for the Fe^2+^/Fe^3+^ half-reaction from −0.77 V to −0.67 V, and the Fe^3+^ is unable to oxidize ascorbic acid [71]. The reaction shown in Eq. (3) is slow, and it is presumed that the chelation of Fe^3+^ by UA slows the reaction even further. Therefore, it is presumed that in reality the reaction shown in Eq. (4) generally proceeds. In this experiment, FeCl_2_ solution was introduced at a constant rate, but indeed, when the addition was stopped, the decrease in UA and production of PA also declined dramatically (data not shown). Therefore, it is thought that this decreased the likelihood that the reaction described by Eq. (3) for recycling Fe^2+^ and producing hydroperoxyl radicals would occur. Furthermore, the coupling of two hydrogen peroxyl radical molecules yields HOOOOH (Eq. (5)), which degrades into H_2_O_2_ and singlet oxygen via a Russell–like mechanism (Eq. (6)) [72].

H_2_O_2_ + Fe^2+^→HO• + HO^−^ + Fe^3+^ (Eq. 1)

(Eq. 3) H_2_O_2_ + Fe^3+^→HOO• + H^+^ + Fe^2+^

(Eq. 4) H_2_O_2_ + HO•→HOO• + H_2_O

(Eq. 5) HOO• + HOO•→HOOOOH

(Eq. 6) HOOOOH →^1^O_2_ + H_2_O_2_

In contrast to the above results, di–^18^O-labeled PA and DIAA were also detected in oxidation experiments conducted in H_2_^18^O, indicating that the observed [18][^1^O_2_] was generated from water (Fig. 4C and D). Similarly, non-labeled PA and DIAA were formed during oxidation of UA in H_2_^18^O_2_ (Fig. 5A and B). If a hydroxyl radical molecule absorbs a hydrogen atom from H_2_O (Eq. (7)), H_2_O and H_2_O_2_ can exchange an oxygen atom (Eqs. (Eq. 8), (Eq. 9))). This generation of oxygen–exchanged H_2_O_2_ leads to the formation of ^18^O-labeled or non-labeled singlet oxygen, respectively (Eqs. (1), (3)–(6)).

(Eq. 7) HO• + H_2_O→ + HO•

(Eq. 8) H^16^O• (H^18^O•) + H_2_^18^O (H_2_^16^O)→H_2_^16^O (H_2_^18^O) + H^18^O• (H^16^O•)

(Eq. 9) H^18^O• (H^16^O•) + H^18^O• (H^16^O•)→H_2_^18^O_2_ (H_2_^16^O_2_)

To determine whether hydroxyl radical reacts with H_2_O to form another hydroxyl radical molecule, Tyr was hydroxylated via the Fenton reaction. Hydroxyl radical is known to react with aromatic compounds, resulting in substitution of a hydroxyl group derived from hydroxyl radical into the aromatic ring. Indeed, formation of HO-Tyr was observed during Fenton reaction–induced Tyr oxidation (Fig. 6A, B, and 6C). Tyr was also oxidized in H_2_^18^O, which resulted in generation of mono–^18^O-labeled Tyr in addition to non-labeled HO-Tyr (Fig. 6D). Moreover, mono–^18^O-labeled hydroxyl salicylic acid was produced when salicylic acid was oxidized in H_2_^18^O, thus providing further confirmation (data not shown). These results strongly suggest that ^16^O-labeled hydroxyl radical formed from H_2_^16^O_2_ absorbed a hydrogen atom from H_2_^18^O to produce ^18^O-labeled hydroxyl radical (Eq. (8)).

In order to examine singlet oxygen formation via the Fenton reaction *in vivo*, LPS–initiated pseudo–inflammation in human blood was analyzed. Under inflammatory conditions, the Fenton reaction is believed to occur via H_2_O_2_ formation, thereby inducing significant oxidative stress [[73], [74], [75], [76]]. As expected, OUA, a hydrolysate of PA, increased in LPS–containing human whole blood, whereas no significant changes in OUA concentration were observed in RBC–depleted blood, which was similar to whole blood except for the addition of LPS (Fig. 7B). Hemoglobin has been shown to cause the Fenton reaction by acting as an iron source [77,78]. The present results suggest that singlet oxygen was generated through the hemoglobin-induced Fenton reaction during pseudo-inflammation.

In this paper, we propose that the Fenton reaction system generates singlet oxygen *in vivo*. It is believed that the Fenton reaction normally occurs *in vivo* due to an abundance of iron. Therefore, it is now believed that singlet oxygen is generated *in vivo* more than previously thought. UA, needless to say, is an important water-soluble antioxidant, and it is known to react with not only radical species to eliminate them but also peroxynitrite [79], hypochlorite ions [80], and singlet oxygen [81]. UA is also thought to protect organisms from oxidative stress. In particular, UA is expected to play an important role in protecting nerves. Patients with Alzheimer's disease [82], Parkinson's disease [83,84], and amyotrophic lateral sclerosis (ALS) [85] reportedly have lower serum UA levels than healthy individuals; however, gout patients reportedly have a lower risk of developing Alzheimer's disease [86], Parkinson's disease, multiple sclerosis, or ALS [87]. Therefore, it is highly likely that the antioxidant activity specific to UA plays an important role in these neurological diseases. On the other hand, it has also been reported that PA levels increase in the cerebrospinal fluid after hemorrhage in severe subarachnoid hemorrhage [88]. Because PA was identified as a singlet oxygen–specific oxidation product of UA, a large amount of singlet oxygen is thought to be generated under such conditions [66,67]. Thus, it is speculated that recognition of the biological importance of the antioxidant activity of UA against singlet oxygen will increase in the future.

## 5 Conclusion

In this study, we demonstrated that the Fenton reaction between H_2_O_2_ and Fe^2+^, which is considered a conventional hydroxyl radical–generating reaction, predominantly produces ^1^O_2_ addition to hydroxyl radical. Therefore, we believe the results of previous studies related to the Fenton reaction should be reconsidered. We found that the ^1^O_2_ generated in our study was derived from H_2_O_2_ independent of dissolved oxygen. The underlying mechanism was postulated to involve the decomposition of HOOOOH formed by the coupling of two hydrogen peroxyl radical molecules.

Indeed, we demonstrated that blood levels of OUA, which is considered a singlet oxygen marker for singlet oxygen production [70], were elevated during LPS–induced pseudo-inflammation, although OUA levels did not increase significantly in RBC-depleted blood. Therefore, we propose a novel singlet oxygen–generating function of the Fenton reaction, which is primarily known as a conventional hydroxyl radical–production pathway *in vivo*.

## CRediT authorship contribution statement

**Rino Shimizu:** Investigation, Methodology, Validation. **Haruki Watanabe:** Investigation. **Sayaka Iida:** Investigation, Validation. **Yorihiro Yamamoto:** Writing – review & editing. **Akio Fujisawa:** Conceptualization, Project administration, Resources, Supervision, Writing – original draft.

## Statement of Ethics

The protocol of this study was approved by the Ethics Committee of Tokyo University of Technology (E18HS-022).

## Funding Source

We have no funding source for this work.

## Declaration of competing interest

We have not received any financial support or other benefits from commercial sources for the work reported in this manuscript. None of the authors have financial interests that could create potential conflicts of interest or the appearance of a conflict of interest with regard to this work.
